# Supplementary material for: clrDV: a differential variability test for RNA-Seq data based on the skew-normal distribution
Source: PeerJ. 2023 Sep 29;11:e16126. doi: 10.7717/peerj.16126 (PMC10544356; doi:10.7717/peerj.16126)
Supplement: Supplemental Information 1 [file peerj-11-16126-s001.pdf]

Supplementary Material for "clrDV: A Differential Variability Test for RNA-Seq Data Based on the Skew-normal Distribution"

Hongxiang Li[1] and Tsung Fei Khang[1,2]

[1]**Institute of Mathematical Sciences, Faculty of Science, Universiti Malaya, 50603 Kuala Lumpur, Malaysia**
[2]**Universiti Malaya Centre for Data Analytics, Universiti Malaya, 50603 Kuala Lumpur, Malaysia**

S1 THE SKEW-NORMAL DISTRIBUTION

S1.1 The original form of the skew-normal distribution

Let $\phi(z)$ be the standard normal probability density function $\phi(z) = \frac{1}{\sqrt{2\pi}}e^{-\frac{z^2}{2}}$, with cumulative distribution function $\Phi(z) = \int_{-\infty}^{z} \phi(t)\,dt$. The probability density function of the skew-normal distribution is given by

$$\varphi(z,\alpha) = 2\phi(z)\Phi(\alpha z),$$

where $\alpha \in \mathbb{R}$ is the skewness parameter. Suppose Z is a random variable that has a skew-normal distribution (i.e. $Z \sim \mathrm{SN}(\alpha)$). Its mean and its variance are given by $\mu_z = \mathbb{E}(Z) = b\delta, \quad \sigma_z^2 = \mathrm{Var}(Z) = 1-(b\delta)^2$, respectively, where $b = \sqrt{2/\pi}$ and $\delta = \alpha/\sqrt{1+\alpha^2}$. The formal derivation of the properties of the skew-normal distribution is due to Azzalini (1985) who treated the skew-normal distribution as a generalization of the normal distribution. Historically, the skew-normal model was arrived at by several different authors in other contexts (e.g. as a prior distribution in Bayesian analysis by O'Hagan and Leonard (1976); see Azzalini (2022)). However, these authors did not elaborate further on the theoretical properties of the skew normal as in Azzalini (1985).

S1.2 The skew-normal distribution - direct and centered parametrizations

The probability density function of a skew-normal distribution with direct parameters (DP) is given by

$$f(y_{gi};\xi_g,\omega_g,\alpha_g) = \frac{2}{\omega_g}\phi\left(\frac{y_{gi}-\xi_g}{\omega_g}\right)\Phi\left(\alpha_g\frac{y_{gi}-\xi_g}{\omega_g}\right),$$

with location parameter $\xi_g \in \mathbb{R}$, scale parameter $\omega_g \in \mathbb{R}^+$, and skewness parameter $\alpha_g \in \mathbb{R}$; $\phi(\cdot)$ and $\Phi(\cdot)$ are the probability density function and the cumulative distribution function of the standard normal distribution, respectively. The skew-normal distribution

with centered parameters (CP) is derived from the DP form via the mapping (Azzalini and Capitanio, 2014)

$$\mu_g = \xi_g + b\omega_g\delta_g,\ \sigma_g = \omega_g\sqrt{1-b^2\delta_g^2},\ \gamma_g = \frac{4-\pi}{2}\frac{b^3\alpha_g^3}{\left\{1+(1-b^2)\alpha_g^2\right\}^{3/2}}; \tag{1}$$

and the inverse mapping is provided by

$$\xi_g = \mu_g - b\omega_g\delta_g, \quad \omega_g = \frac{\sigma_g}{\sqrt{1-b^2\sigma_g^2}}, \quad \alpha_g = \frac{R}{\sqrt{b^2-(1-b^2)R^2}}, \tag{2}$$

where $b = \sqrt{2/\pi}$, $\delta_g = \alpha_g/\sqrt{1+\alpha_g^2}$, and $R = \sqrt[3]{2\gamma_g/(4-\pi)}$.

For a single sample, the log-likelihood function for $\boldsymbol{\theta}_g^{(D)} = (\xi_g, \omega_g, \alpha_g)^T$ is given by

$$\ell_1 = \log L(\boldsymbol{\theta}_g^{(D)}; y_{gi}) = c - \log\omega_g - \frac{(y_{gi}-\xi_g)^2}{2\omega_g^2} + \zeta_0\left(\alpha_g\frac{y_{gi}-\xi_g}{\omega_g}\right),$$

where c is a constant and $\zeta_0(\cdot) = \log\left\{2\Phi(\cdot)\right\}$. Taking $z_{gi} = (y_{gi}-\xi_g)/\omega_g$, we obtain the partial derivatives of ℓ_1:

$$\frac{\partial\ell_1}{\partial\xi_g} = \frac{z_{gi}}{\omega_g} - \frac{\alpha_g}{\omega_g}\zeta_1(\alpha_g z_{gi}),\ \frac{\partial\ell_1}{\partial\omega_g} = -\frac{1}{\omega_g} + \frac{z_{gi}^2}{\omega_g} - \frac{\alpha_g}{\omega_g}\zeta_1(\alpha_g z_{gi})z_{gi},\ \frac{\partial\ell_1}{\partial\alpha_g} = \zeta_1(\alpha_g z_{gi})z_{gi};$$

thus the likelihood equations for a sample of size n are given by

$$\sum_{i=1}^n z_{gi} - \alpha_g\sum_{i=1}^n \zeta_1(\alpha_g z_{gi}) = 0,\ \sum_{i=1}^n {z_{gi}}^2 - \alpha_g\sum_{i=1}^n z_{gi}\zeta_1(\alpha_g z_{gi}) = n,\ \sum_{i=1}^n z_{gi}\zeta_1(\alpha_g z_{gi}) = 0, \tag{3}$$

where $\zeta_1(\cdot) = \phi(\cdot)/\Phi(\cdot)$. Numerical methods are necessary to solve these equations. Azzalini and Capitanio (2014) suggested that a sample size up to about 50 may be necessary for the skew- normal distribution. To initialize the search, method of moments (MM) estimates are chosen as starting points for the CP components in Equation (1). The MM estimators for the centered parameters are given by

$$\tilde{\mu}_g = \bar{Y}_g, \quad \tilde{\sigma}_g = S_g, \quad \tilde{\gamma}_g = \frac{M_{g,3}}{S_g^3}, \tag{4}$$

respectively, where $\bar{Y}_g$ is the sample mean, S_g is the sample standard deviation, and $M_{g,3}$ is the sample third central moment. By estimating the CP components in Equation (1) using Equation (4), and then converting them to DP components using Equation (2), we obtain the MM estimators of the DP components: $\bar{\xi}_g, \bar{\omega}_g$ and $\bar{\alpha}_g$. Subsequently, a search of the DP space where Equation (3) holds is done. Once $\hat{\boldsymbol{\theta}}_g^{(D)} = (\hat{\xi}_g, \hat{\omega}_g, \hat{\alpha}_g)$ is obtained, it is mapped to Equation (1) to get $\hat{\boldsymbol{\theta}}_g^{(C)} = (\hat{\mu}_g, \hat{\sigma}_g, \hat{\gamma}_g)$, the maximum likelihood estimators of the centered parameters.

Under regular maximum likelihood estimation, certain data values can produce a divergent $\hat{\alpha}_g$. To overcome this problem, Azzalini and Arellano-Valle (2013) proposed

a maximum penalized likelihood estimation ("Qpenalty") approach. A non-negative penalty term Q that penalizes the divergence of the skewness parameter α_g is formulated as $Q = c_1 \log(1 + c_2\alpha_g^2)$, where $c_1 \approx 0.87591$ and $c_2 \approx 0.85625$ (Azzalini and Arellano-Valle, 2013; Azzalini and Capitanio, 2014). Then, the maximum penalized likelihood for $\boldsymbol{\theta}_g^{(D)}$ is the penalized log-likelihood

$$\ell_p(\boldsymbol{\theta}_g^{(D)}) = \ell(\boldsymbol{\theta}_g^{(D)}; \boldsymbol{y}_g) - Q, \tag{5}$$

where $\boldsymbol{y}_g = (y_{g1}, y_{g2}, \ldots, y_{gn})$, $\ell(\boldsymbol{\theta}_g^{(D)}; \boldsymbol{y}_g)$ is the log- likelihood function with respect to the parameter vector $\boldsymbol{\theta}_g^{(D)}$:

$$\ell(\boldsymbol{\theta}_g^{(D)}; \boldsymbol{y}_g) = \text{constant} - n\log\omega_g - \sum_{i=1}^{n} \frac{(y_{gi} - \xi_g)^2}{2\omega_g^2} + \sum_{i=1}^{n} \zeta_0\big(\alpha_g \frac{y_{gi} - \xi_g}{\omega_g}\big).$$

The maximum penalized likelihood estimator (MPLE), $\tilde{\boldsymbol{\theta}}_g^{(D)}$, is a finite point that maximizes $\ell_p(\boldsymbol{\theta}_g^{(D)})$. The standard errors of $\tilde{\boldsymbol{\theta}}_g^{(D)}$ can be approximated from the corresponding penalized information matrix as $\text{Var}(\tilde{\boldsymbol{\theta}}_g^{(D)}) \approx -\ell_p''(\tilde{\boldsymbol{\theta}}_g^{(D)})^{-1}$.

The "MPpenalty" approach (Azzalini and Capitanio, 2014) defines the penalty function Q in Equation (5) as $-\log \pi_m(\alpha_g)$, where π_m is a prior distribution for the skewness parameter α_g. The matching prior (Cabras et al., 2012) for α_g, allowing for the presence of $\boldsymbol{\psi} = (\xi_g, \omega_g)$, is given by

$$\pi_m(\alpha_g) \propto \big(I_{\alpha_g\alpha_g}(\hat{\boldsymbol{\psi}}, \alpha_g) - I_{\alpha_g\boldsymbol{\psi}}(\hat{\boldsymbol{\psi}}, \alpha_g) I_{\boldsymbol{\psi}\boldsymbol{\psi}}(\hat{\boldsymbol{\psi}}, \alpha_g)^{-1} I_{\boldsymbol{\psi}\alpha_g}(\hat{\boldsymbol{\psi}}, \alpha_g)\big)^{1/2},$$

where the terms involved are specific blocks of the Fisher information matrix $\boldsymbol{I}$ of $\boldsymbol{\theta}_g^{(D)}$. Since $\pi_m(0) = 0$, the matching prior penalty effectively penalizes $\alpha_g = 0$ with $Q = \infty$.

S1.3 Fisher Information Matrix

For the direct parameters (DP) vector $\boldsymbol{\theta}^{(D)} = (\xi, \omega, \alpha)$, the Fisher information matrix is given by

$$\boldsymbol{I}_{\boldsymbol{\theta}^{(D)}} = \begin{bmatrix} \frac{1+\alpha^2 a_0}{\omega^2} & \frac{1}{\omega^2}\Big(\mathbb{E}(Z)\frac{1+2\alpha^2}{1+\alpha^2} + \alpha^2 a_1\Big) & \frac{1}{\omega}\Big\{\frac{b}{(1+\alpha^2)^{3/2}} - \alpha a_1\Big\} \\ \frac{1}{\omega^2}\Big(\mathbb{E}(Z)\frac{1+2\alpha^2}{1+\alpha^2} + \alpha^2 a_1\Big) & \frac{2+\alpha^2 a_1}{\omega^2} & -\frac{\alpha a_2}{\omega} \\ \frac{1}{\omega}\Big\{\frac{b}{(1+\alpha^2)^{3/2}} - \alpha a_1\Big\} & -\frac{\alpha a_2}{\omega} & a_2 \end{bmatrix},$$

where $b = \sqrt{\pi/2}$, $a_k = a_k(\alpha) = \mathbb{E}\Big(Z^k \zeta_1^2(\alpha Z)\Big)$, $\zeta_1(\cdot) = \phi(\cdot)/\Phi(\cdot), k = 0, 1, 2$ (Azzalini, 1985). The matrix $I_{\boldsymbol{\theta}^{(D)}}$ becomes singular as $\alpha \to 0$. This problem prevents the direct application of maximum likelihood estimation (MLE) for estimating the parameters of the DP form of the skew-normal distribution. In the same paper, Azzalini (1985) introduced the centered parametrization form to address the singulariy problem. He redefines a skew-normal variable

$$Y = \mu + \sigma\frac{Z - \mu_z}{\sigma_z},$$

which has $\mathbb{E}(Y) = \mu$ and $\text{Var}(Y) = \sigma^2$. The centered parameters (CP) vector $\boldsymbol{\theta}^{(C)} = (\mu, \sigma, \gamma)$ has parameter space $\mathbb{R} \times \mathbb{R}^+ \times (-0.9953, 0.9953)$ (Azzalini and Capitanio, 2014). The skewness parameter γ is the coefficient of skewness of Z, and also that of Y. Thus, we write $Y \sim \text{SN}_\text{C}(\mu, \sigma, \gamma)$. Figure S1 shows examples of the probability density functions of the skew-normal distribution with CP for different values of μ, σ and γ. For centered parameters $\boldsymbol{\theta}^{(C)} = (\mu, \sigma, \gamma)$, the Fisher information matrix is given by

$$\boldsymbol{I}_{\boldsymbol{\theta}^{(C)}} = \boldsymbol{D}^T \boldsymbol{I}_{\boldsymbol{\theta}^{(D)}} \boldsymbol{D},$$

where $\boldsymbol{D}$ is the Jacobian matrix, that is, the derivatives of the parameters $\boldsymbol{\theta}^{(D)} = (\xi, \omega, \alpha)$ with respect to $\boldsymbol{\theta}^{(C)} = (\mu, \sigma, \gamma)$. The Jacobian matrix $\boldsymbol{D}$ is given by

$$\boldsymbol{D} = \begin{bmatrix} 1 & -\frac{\mu_z}{\sigma_z} & \frac{\partial}{\partial \gamma}\xi \\ 0 & \frac{1}{\sigma_z} & \frac{\partial}{\partial \gamma}\omega \\ 0 & 0 & \frac{\partial}{\partial \gamma}\alpha \end{bmatrix},$$

where $\mu_z = b\delta$ and $\sigma_z^2 = 1 - b^2\delta^2$. The terms in the last column of $\boldsymbol{D}$ are given by

$$\frac{\partial \xi}{\partial \gamma} = -\frac{\sigma \mu_z}{3\sigma_z \gamma}, \quad \frac{\partial \omega}{\partial \gamma} = -\frac{\sigma}{\sigma_z^2}\frac{d\sigma_z}{d\alpha}\frac{d\alpha}{d\gamma}, \quad \frac{\partial \alpha}{\partial \gamma} = \frac{2}{3(4-\pi)}\left(\frac{1}{TR^2} + \frac{1-b^2}{T^3}\right),$$

where

$$\frac{d\sigma_z}{d\alpha} = -\frac{\mu_z}{\sigma_z}\frac{b}{(1+\alpha^2)^{3/2}}, \; T = \left\{b^2 - (1-b^2)R^2\right\}, \text{ and } R = \sqrt[3]{\frac{2\gamma}{4-\pi}}.$$

After some algebra, $\boldsymbol{I}_{\boldsymbol{\theta}^{(C)}}$ can be expressed as

$$\boldsymbol{I}_{\boldsymbol{\theta}^{(C)}} = \begin{bmatrix} \left(2 + \gamma^2 a_2\right)/\sigma^2 & \left\{b\delta\frac{1+2\gamma^2}{1+\gamma^2} + \gamma^2 a_1\right\}/\sigma^2 & -\gamma a_2/\sigma \\ \left\{b\delta\frac{1+2\gamma^2}{1+\gamma^2} + \gamma^2 a_1\right\}/\sigma^2 & \left(1 + \gamma^2 a_0\right)/\sigma^2 & \left\{\frac{b}{(1+\gamma^2)^{3/2}} - \gamma a_1\right\}/\sigma \\ -\gamma a_2/\sigma & \left\{\frac{b}{(1+\gamma^2)^{3/2}} - \gamma a_1\right\}/\sigma & a_2 \end{bmatrix},$$

where

$$b = \sqrt{2/\pi}, \; \delta = \gamma/\sqrt{1+\gamma^2} \text{ and } a_k = a_k(\gamma) = \int_{\mathbb{R}} 2z^k \frac{\phi(\gamma z)\phi(z)}{\Phi(\gamma z)} dz, \; k = 0, 1, 2,$$

following the notation in Liseo and Loperfido (2006). The Fisher information matrix $\boldsymbol{I}_{\boldsymbol{\theta}^{(C)}}$ converges to a diagonal matrix with diagonal $(1/\sigma^2, 2/\sigma^2, 1/6)$, as $\gamma \to 0$ (Azzalini, 1985; Azzalini and Capitanio, 2014).

S2 LIST OF R PACKAGES USED

The following R packages (according to alphabetical order) were used in the present work: `compositions` (van den Boogaart et al., 2022), `DiffDist` (Roberts, 2023), `edgeR` (Robinson et al., 2010), `gamlss` (Rigby and Stasinopoulos, 2005), `gridExtra` (Baptiste, 2017), `httr` (Wickham, 2022), `jsonlite` (Ooms, 2014), `MDSeq` (Ran and Daye, 2017), `missMethyl` (Phipson and Oshlack, 2014), `polyester` (Alyssa et al., 2022), `readr` (Wickham et al., 2022), `sn` (Azzalini, 2022), `VennDiagram` (Chen, 2022) and `vioplot` (Adler and Kelly, 2020).

S3 CAPTIONS FOR SUPPLEMENTARY TABLES

Table S3: List of DV genes detected by clrDV for the control vs. AD comparison in the analysis of the Mayo RNA-Seq dataset.
Table S4: List of DV genes detected by clrDV for the control vs. PSP comparison in the analysis of the Mayo RNA-Seq dataset.
Table S5: List of DV genes detected by clrDV, MDSeq, and GAMLSS for the control vs. AD comparison in the analysis of the Mayo RNA-Seq dataset.
Table S6: List of DV genes detected by clrDV, MDSeq, and GAMLSS for the control vs. PSP comparison in the analysis of the Mayo RNA-Seq dataset.

REFERENCES

Adler, D. and Kelly, S. T. (2020). *vioplot: violin plot*. R package version 0.3.7.

Alyssa, C. F., Andrew, E. J., Rory, K., and Jeffrey, T. L. (2022). *polyester: Simulate RNA-seq reads*. R package version 1.32.0.

Azzalini, A. (1985). A class of distributions which includes the normal ones. *Scand J Stat.*, 12(2):171–178.

Azzalini, A. (2022). *The R package `sn`: The skew-normal and related distributions such as the skew-t and the SUN (version 2.1.0)*. Università degli Studi di Padova, Italia. Home page: `http://azzalini.stat.unipd.it/SN/`.

Azzalini, A. and Arellano-Valle, R. B. (2013). Maximum penalized likelihood estimation for skew-normal and skew-t distributions. *Journal of Statistical Planning and Inference*, 143(2):419–433.

Azzalini, A. and Capitanio, A. (2014). *The Skew-Normal and Related Families*. Cambridge University Press, Cambridge.

Baptiste, A. (2017). *gridExtra: Miscellaneous Functions for "Grid" Graphics*. R package version 2.3.

Cabras, S., Racugno, W., Castellanos, M. E., and Ventura, L. (2012). A matching prior for the shape parameter of the skew-normal distribution. *Scandinavian Journal of Statistics*, 39(2):236–247.

Chen, H. (2022). *VennDiagram: Generate High-Resolution Venn and Euler Plots*. R package version 1.7.3.

Liseo, B. and Loperfido, N. (2006). A note on reference priors for the scalar skew-normal distribution. *J Stat Plann Infer.*, 136(2):373–389.

O'Hagan, A. and Leonard, T. (1976). Bayes estimation subject to uncertainty about parameter constraints. *Biometrika*, 63(1):201–203.

Ooms, J. (2014). The jsonlite Package: A Practical and Consistent Mapping Between JSON Data and R Objects. *arXiv:1403.2805 [stat.CO]*.

Phipson, B. and Oshlack, A. (2014). DiffVar: a new method for detecting differential variability with application to methylation in cancer and aging. *Genome Biol.*, 15(9):1–16.

Ran, D. and Daye, Z. J. (2017). *MDSeq: Gene expression mean and variability analysis for RNA-seq counts*. R package version 1.0.5.

Rigby, R. A. and Stasinopoulos, D. M. (2005). Generalized additive models for location, scale and shape. *J R Stat Soc C-Appl.*, 54(3):507–554.

Roberts, A. G. K. (2023). *DiffDist: Hierarchical Mixture Model for RNA-seq Differential Distribution Testing*. R package version 0.1.0.9000.

Robinson, M. D., McCarthy, D. J., and Smyth, G. K. (2010). edgeR: a Bioconductor package for differential expression analysis of digital gene expression data. *Bioinformatics*, 26(1):139–140.

van den Boogaart, K. G., Tolosana-Delgado, R., and Bren, M. (2022). *compositions: Compositional Data Analysis*. R package version 2.0-4.

Wickham, H. (2022). *httr: Tools for Working with URLs and HTTP*. R package version 1.4.4.

Wickham, H., Hester, J., and Bryan, J. (2022). *readr: Read Rectangular Text Data*. R package version 2.1.2.

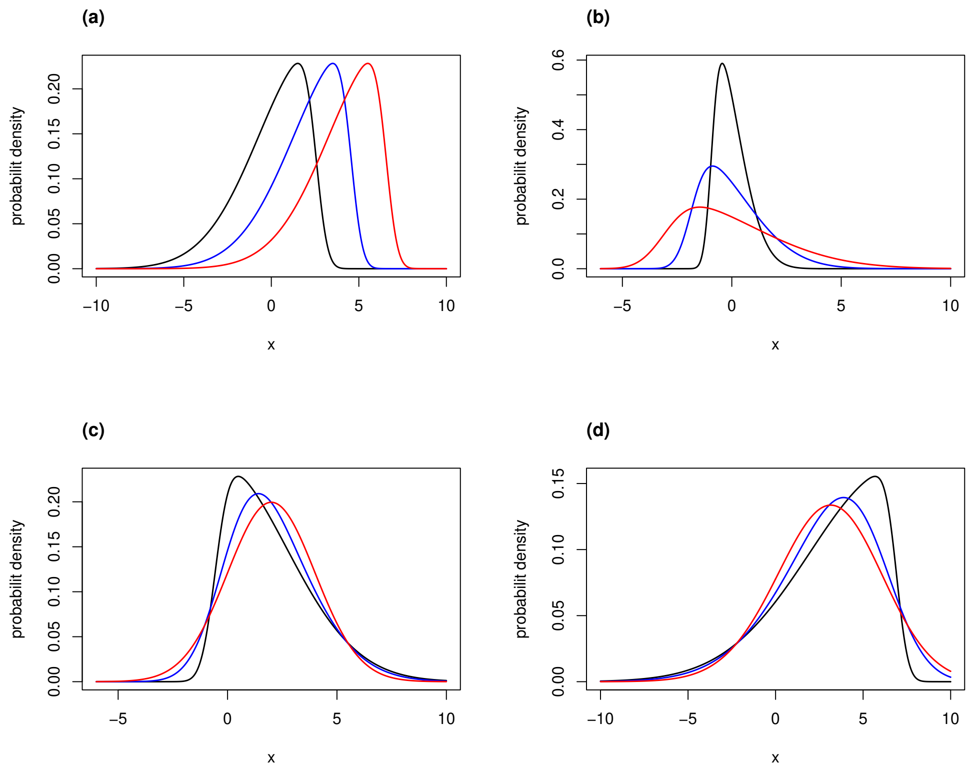

Figure S1. Probability density functions of the skew-normal distribution with centered parameters. (a) $\sigma = 2$, $\gamma = -0.9$; black: $\mu = 0$, blue: $\mu = 2$, red: $\mu = 4$; (b) $\mu = 0$, $\gamma = 0.8$; black: $\sigma = 0.75$, blue: $\sigma = 1.5$, red: $\sigma = 2.5$; (c) $\mu = 2$, $\sigma = 2$; black: $\gamma = 0.9$, blue: $\gamma = 0.5$, red: $\gamma = 0$; and (d) $\mu = 3$, $\sigma = 3$; black: $\gamma = -0.95$, blue: $\gamma = -0.5$, red: $\gamma = -0.1$.

Table S1. Mean computing times (in seconds) for each of the five DV tests applied to data simulated from the Valentim dataset (30 instances). Standard deviation in parentheses. BH and BY variants of GAMLSS have similar computing time.

Sample size	Method				
per group	clrDV	MDSeq	diffVar	GAMLSS	DiffDist
50	81(5)	59(2)	0.2(0.01)	79(1)	221(4)
100	69(11)	103(10)	0.4(0.2)	97(12)	422(8)
150	67(2)	143(7)	0.7(0.3)	116(1)	690(16)
200	76(8)	196(14)	1.3(0.4)	153(16)	922(7)

Table S2. Mean computing times (in seconds) for each of the five DV tests applied to data simulated from the Kelmer dataset (30 instances). Standard deviation in parentheses. BH and BY variants of GAMLSS have similar computing time.

Sample size	Method				
per group	clrDV	MDSeq	diffVar	GAMLSS	DiffDist
50	73(5)	32(1)	0.2(0.02)	62(1)	228(1)
100	53(2)	45(2)	0.4(0.1)	70(3)	427(7)
150	58(2)	63(3)	0.7(0.3)	81(2)	634(15)
200	65(1)	84(4)	1.0(0.3)	95(1)	864(18)
